# Intersections of endocrine pathways and the epithelial mesenchymal transition in endometrial cancer

**DOI:** 10.3389/fonc.2022.914405

**Published:** 2022-08-16

**Authors:** Julia H. Gelissen, Gloria S. Huang

**Affiliations:** Department of Obstetrics, Gynecology, and Reproductive Sciences, Yale University, New Haven, CT, United States

**Keywords:** EMT, endometrial cancer, endocrine, estrogen, diabetes, glucose, metformin

## Abstract

The epithelial mesenchymal transition (EMT) is the process by which cancer cells of epithelial origin, including endometrial cancer, acquire a mesenchymal phenotype with enhanced migratory and invasive capacity, to facilitate metastasis. The regulation of EMT is tissue-specific, and in endometrial cancer, endocrine signaling pathways serve as critical regulators of EMT. The intersections of endocrine signaling and EMT highlight potential avenues for therapeutic intervention to target cancer metastasis with the aim of reduced mortality.

## Introduction

Worldwide endometrial cancer (EC) incidence and mortality is rising. EC is the most common gynecologic cancer in the United States, with an estimated 66,570 new cases in 2021. Though the overall prognosis for endometrial cancer is favorable with an 81.1% 5-year relative survival rate, prognosis varies greatly by disease stage. Those with uterine-confined malignancy have a 94.9% 5-year survival rate, while those with distant disease have a 17.8% 5-year survival (1). These data underscore the impact of tumor metastasis on endometrial cancer mortality.

The epithelial mesenchymal transition (EMT) is a process known to play an essential role in normal human development and healing, as well as in cancer metastasis. Through the EMT, cells lose their typical intercellular connections and exhibit increased motility, invasiveness, and stem cell-like properties, including self-renewal, that facilitate metastasis (2, 3). EMT has been categorized into three different types to describe its role in physiologic and pathologic processes. Type I EMT contributes to key steps in embryogenesis including gastrulation and neural crest formation, type II EMT is induced in response to inflammation and contributes to wound healing, and type III EMT describes the role of EMT in promoting cancer metastasis (4). This process is hallmarked by the loss of E-cadherin, a key component in cellular adherens junctions, and an increase in mesenchymal cell markers, including N-cadherin, vimentin, and fibronectin, and matrix metalloproteinases (MMPs). The change from expression of epithelial to mesenchymal markers is driven by upregulation of certain transcription factors, including Zinc finger E-box binding homeobox family proteins (ZEB1 and ZEB2), Snail family proteins Snail1 (SNAIL) and Snail2 (SLUG), and Twist family proteins Twist1 and Twist2 (4), which have been shown to directly repress the expression of E-cadherin (5–9). Additionally, the TGF-β, WNT, and PI3K signaling pathways are all known to play an important role inducing EMT (10, 11).

The EMT phenotype in endometrial cancer is characterized by the expression of EMT-associated transcription factors, loss of epithelial cell markers and acquisition of mesenchymal cell markers. Endometrial cancer EMT is associated with expression of the EMT-associated transcription factors SNAIL, SLUG, TWIST2, ZEB1 and ZEB2 (12) and decreased expression of E-cadherin (13, 14). Moreover, EMT has been found to have clinical and prognostic implications in endometrial cancer. ZEB2, SNAIL, and SLUG expression have each been associated with aggressive clinical characteristics, such as higher stage, grade, non-endometrioid histology, deep myometrial invasion, positive peritoneal cytology, lymph node involvement, and distant metastases (12, 14) and SLUG expression is associated with decreased 5-year survival (12). Reduced expression of E-cadherin is associated with increased grade, stage, non-endometrioid histology, deep myometrial invasion, and positive peritoneal cytology in endometrial cancer (14), and EMT status is a significant predictor of lower overall survival in EC (14).

As a malignancy arising from a hormonally-responsive reproductive organ, endometrial cancer is linked to multiple endocrinological perturbations including unopposed estrogen, obesity, insulin resistance and diabetes (15–18). Connections between these pathways and the epithelial mesenchymal transition in endometrial cancer underscore the complex and interrelated processes that contribute to the development and progression of endometrial cancer. Herein, we describe evidence for endocrine regulation of the epithelial mesenchymal transition of endometrial cancer, and remaining knowledge gaps for future investigation.

## Endocrine pathways in the EMT of endometrial cancer

### Sex hormones

The normal endometrium is a sex hormone responsive tissue, and unopposed estrogen exposure is a recognized driver of endometrial carcinogenesis (15). It may be unsurprising that there is ample evidence supporting the role of estrogen, estrogen-related compounds, and progesterone in the EMT of endometrial cancer.

#### Estrogen

Estrogen drives the physiologic growth and proliferation of endometrial cells during the menstrual cycle. Unopposed estrogen has been shown to cause endometrial hyperplasia and pre-cancerous changes in animal models (19) and increase the risk for invasive cancer in humans (15). In addition to being implicated in the development of endometrial cancer, estrogen stimulation has been shown to cause tumor growth, migration and invasion in endometrial cancer cell lines (20–24) and higher levels of circulating estrogen have been associated with recurrence in endometrioid endometrial cancers (25). Evidence further supporting estrogen’s role in the EMT of endometrial cancer include the increased expression of the transcriptions factors SNAIL, SLUG, and ZEB2 and mesenchymal markers N-cadherin and Vimentin, the decreased expression of the epithelial marker E-cadherin and activation of the PI3K and ERK signaling pathways in endometrial cancer cell lines treated with estradiol (20–22, 24, 26). Estrogen has also been shown to contribute to EMT through the ubiquitin-proteasome pathway. Estrogen upregulates UBE2C, a ubiquitin-conjugating enzyme, likely through ERα signaling, leading to an EMT-phenotype (increased cell proliferation, migration, and invasion; increased vimentin and decreased E-cadherin) through downregulation of the tumor suppressor p53 and its target p21 (21).

In addition to estrogen’s direct role on endometrial cancer cells, the tumor microenvironment also regulates estrogen-dependent EMT. Normal endometrial stromal cells can reduce cell growth, induce apoptosis, inhibit estradiol-induced migration and invasion, and reverse estradiol-induced mesenchymal marker expression in endometrial cancer cell lines (22). However, ERα+ macrophages in the tumor microenvironment can have the opposite effect. Conditioned medium from agonist-treated ERα+ M2 macrophages increased endometrial cancer cell migration, invasion and irregular cell morphology. It also increased N-cadherin, Vimentin, and TWIST1 and decreased E-cadherin. These effects appear to be mediated by the chemokine CCL18 activating the PI3K pathway. Supporting their role in driving cancer metastasis, there was a much higher percentage of ERα+ macrophages in advanced stage tumor tissues vs early stage (27).

The orphan nuclear receptor estrogen related receptor alpha (ERRα), which shares DNA sequence homology with ERα, also has a role to play in EMT. Although ERRα does not bind estrogens, it has been shown to engage in cross-talk with estrogen signaling pathways (28) and, in animal studies, its expression in the uterus is stimulated by estrogen as a downstream target of ERα (29). There is evidence that ERRα may mediate the signaling of TGFβ in endometrial cancer associated stromal cells and TGFβ’s subsequent activation of EMT (30). It remains to be answered though if ERRα’s role in EMT through TGFβ is a downstream result of estrogen signaling.

Unlike type I endometrial cancer, type II endometrial cancers have been thought to occur in the absence of unopposed estrogen and classically display an aggressive clinical phenotype. Type II endometrial cancers are also hallmarked by a lack of estrogen and progesterone receptors. Corresponding with this, ERα negativity has been associated with non-endometrioid histology, grade 3 tumor, stage III/IV disease, and worse survival in endometrial cancer (31). These clinical characteristics may be secondary to activation of the EMT in ERα negative cancers, as low mRNA expression of the gene encoding ERα, ESR1, has been associated with high expression of the EMT transcription factors SNAIL, SLUG, TWIST1, ZEB1, and ZEB2. Alternatively, high expression of ESR1 was significantly associated with E-cadherin, α-catenin, and β-catenin mRNA expression. Furthermore, the same study showed associations between ERα-negativity and activation of Sonic Hedgehog, Wnt, TGF-β, and PI3K pathways (31). These data argue for an estrogen-independent mechanism of EMT activation in type II, ERα negative tumors. Other studies, however, suggest that in ERα negative type II endometrial cancer, estrogen continues to stimulate the epithelial mesenchymal transition through the third estrogen receptor, the G-protein coupled estrogen receptor (GPER). A higher prevalence of GPER in the cytoplasm of type II vs type I cell lines, as well in clinically type II vs type I endometrial cancer tissues, has been seen. Estrogen stimulation of GPER has been further shown to activate the ERK and PI3K pathways in a matrix metalloproteinase (MMP) and EGFR dependent fashion *in vitro*, and GPER antagonism has been shown to block estrogen-stimulated tumor growth *in vivo* (20). Additional support for the role of GPER in the EMT of endometrial cancer, comes from studies investigating miR-195, a miRNA believed to target GPER. In endometrial cancer cell lines, miR-195 decreased cell viability, migration and invasion, and increased the expression of tissue inhibitor of metalloproteinase 2 (TIMP2), while decreasing MMP2 and MMP9. miR-195 further decreased phosphorylated PI3K and AKT (32). Taken together, the above data suggest that estrogen plays an important role in the epithelial mesenchymal transition of both type I and type II endometrial cancers, and that loss of ERα and transition to estrogen signaling through GPER may be an important step in EMT for type II cancers

#### Aromatase

Aromatase is the key enzyme responsible for the conversion of androgens to estrogens. Aromatase is typically expressed in a variety of tissues including the ovary, skin, brain, bone, placenta and adipose tissue (33). Higher expression of aromatase has been identified in endometrial cancer tissues, underscoring the importance of estrogens in the development and progression of endometrial cancers as discussed above. Furthermore, obesity is a significant risk factor for endometrial cancer. This may be explained in part by the increased adipose tissue in these patients and the idea that increased synthesis of extraovarian estrogens is correlated with excess body weight (33). Additionally, elevated plasma levels of testosterone and androstenedione, which are converted to estradiol and estrone by aromatase, are associated with increased risk of endometrial cancer (33).

#### Estrogen-like compounds and endocrine disrupting chemicals

Similar to estrogen itself, a variety of estrogen-related compounds have been implicated in the process of endometrial cancer EMT. The selective estrogen receptor modulators tamoxifen and raloxifene have both been shown stimulate GPER-mediated PI3K and ERK signaling in endometrial cancer cell lines (20). Tamoxifen, which is known to cause endometrial cancer, has also been shown to contribute to the EMT of endometrial cancer through its interaction with a family of miRNA, miR-200s, that can inhibit TGFβ-induced EMT by repressing ZEB1/2 (26, 34). Tamoxifen may stimulate EMT in an ERα dependent fashion by increasing c-Myc promoter inhibition of miR-200s, leading to their downregulation and thereby releasing ZEB2 from miR-200 repression (26).

Phytoestrogens, naturally occurring plant-derived compounds that are structurally similar to estrogen also play a role in EMT. Unlike other estrogen-related compounds, there is evidence that, overall, phytoestrogens such as lignans and soy isoflavones may decrease the risk of endometrial cancer (35). The soy-derived phytoestrogen, genistein, has been shown to reverse the epithelial mesenchymal transition in cells from multiple cancer types and it has specifically been shown to reverse estrogen induced EMT in ER-positive ovarian cancer cells (36). Moreover, genistein has been shown to decrease cell proliferation, decrease ERα mRNA expression, increase progesterone receptor (PR) mRNA expression and decrease phosphorylation of AKT in endometrial cancer cell lines (37). Other studies, however, have shown genistein to increase proliferation and cell cycle progression in endometrial cancer cells (38) and activate ERK signaling through GPER in endometrial cancer (20). Some data has suggested that these disparate findings may be secondary to different effects of genistein at varying concentrations (38). Regardless, further investigation into the role of phytoestrogens in the EMT of EC should be conducted.

Additionally, endocrine disrupting chemicals, natural and synthetic compounds that interfere with the endocrine system by altering the normal function of hormones and hormone receptors, can contribute to the EMT of EC (39). The classic endocrine disruptor bisphenol A (BPA) is known to interact with ERα in a manner distinct from other known ER ligands including estradiol (40) and has been hypothesized to contribute to a wide range of reproductive-related disorders. In endometrial cancer, BPA been shown to induce EMT *in vitro* by increasing cell growth, migration, invasion, mesenchymal cell morphology and mesenchymal markers (41). Similarly, the common herbicide glyphosphate has been shown in some studies to induce ERα signaling in a ligand-independent manner (42) and, in animal models, glyphosphate based herbicides have been shown to induce endometrial hyperplasia and increase endometrial sensitivity to estradiol (43), though glyphosphate’s endocrine-disrupting properties are still debated. Glyphosphate has been shown to induce EMT in endometrial cancer cell lines by increasing cell migration, invasion, and expression of E-cadherin in an estrogen receptor dependent fashion (23). Additional endocrine disrupting chemicals, including phthalates (chemicals often added to plastics and commonly found in household goods, food and cosmetics), dioxins (including 2,3,7,8-Tetrachlorodibenzo-p-dioxin [TCDD] – persistent organic pollutants produced through natural and industrial processes), and triclosan (5-chloro-2-(2,4-dichloro-phenoxy)-phenol – an antimicrobial added to many common hygiene products) have been shown to induce EMT in estrogen dependent non-endometrial cancer cell lines (39). They may therefore be relevant in the process of EMT in EC, though further investigation is required to confirm this. It is clear from these studies that the process of hormonally-mediated EMT in endometrial cancer goes far beyond classic ERα signaling in endometrial glandular cells, and that a variety of common chemical, environmental and food-based inputs may be contributing to endometrial cancer progression. and metastasis in the modern day.

#### Progesterone

Progesterone plays an important regulatory role in endometrial growth and differentiation throughout the menstrual cycle and progestogens are often used in clinical practice to treat abnormal uterine bleeding by thinning the endometrial lining. Along with estrogen receptor positivity, progesterone receptor positivity in endometrial cancer is classically associated with type I cancers. Lack of progesterone receptor in endometrial cancer is associated with poorer prognostic factors, including high tumor grade, non-endometrioid histology and deep myometrial invasion (44, 45). Furthermore, there is evidence that in the normal menstrual cycle and in endometrial cancer, progesterone may increase the expression of the WNT/β-catenin pathway inhibitors DKK1 and FOXO1. It is hypothesized that some of progesterone’s inhibitory effect on endometrial hyperplasia and endometrial cancer may be mediated through its ability to inhibit the WNT/β-catenin pathway (46). The progestogen medroxyprogesterone acetate has also been shown to decrease migration, invasion, and vimentin expression in endometrial cancer cells expressing progesterone receptor B or progesterone receptors A and B, though not in those expressing progesterone receptor A alone. In the same study, progesterone was shown to downregulate a number of signaling pathways known to play an important role in the EMT of endometrial cancer, including PI3K, TGFβ, IGF-1, and EGF (47). Progesterone’s protective role in endometrial cancer may therefore be mediated through its inhibition of EMT in type I EC tissues that retain the progesterone receptor.

### Metabolic disturbances

Obesity and its comorbid condition, diabetes, are significant risk factors for the development of endometrial cancer. It is thought that hyperglycemia, insulin resistance and subsequent increased activity of the insulin/IGF pathway drive the risk of endometrial cancer in this population (16).

#### Insulin and type-1 insulin like growth factor receptor

Insulin and insulin like growth factors (IGFs) play an important role in normal metabolism, and several studies have linked aberrant insulin and IGF signaling to EC development and progression. In endometrial hyperplasia and endometrial cancer, the expression of insulin receptors A and B (IR-A and IR-B) and type-1 insulin like growth factor receptor (IGF-1R) is upregulated compared to normal endometrium (48). IGF-1R overexpression and activation may contribute to endometrial carcinogenesis by decreasing apoptosis through PI3K/AKT pathway signaling (49). Studies of miR-424, a micro RNA that targets and suppresses IGF-1R, show that it is decreased in endometrial cancer cells. Expression of miR-424 is associated with epithelial cellular morphology, increased expression of E-cadherin and decreased expression of vimentin, while decreased expression of miR-424 is associated with increased proliferation and migration in endometrial cancer cells. Clinical data supports this role for IGF-1R in EMT, as patients with lower expression of miR-424 were more likely to have lymph node metastases and higher stage disease (50). IGF-1R is also known to form a hybrid receptor, IR-A/IGF-1R, with the insulin receptor, and this hybrid receptor has been shown to activate the EMT in endometrial cancer. Insulin and IGF-1 are each able to induce the expression of EMT biomarkers in endometrial cancer cell lines (51). Knockdown of IR-A or IGF-1R decreases cell migration and invasion, increases apoptosis, and induces epithelial-type biomarker expression (increased E-cadherin and decreased MMP2, MMP9, N-cadherin, vimentin, fibronectin) through PI3K and ERK signaling. Interestingly, knockdown of both receptors produced these effects most dramatically (51). Clinical data supports the importance of the hybrid insulin and IGF-1 receptor as expression of its activated form, pIGF-1R/pIR, is associated with recurrence in endometrioid endometrial cancers (25). In addition to IGF-1R’s individual effect on endometrial cancer, the roles of IGF-1R and sex hormone signaling in EMT are likely closely interwoven as estrogen has been shown to stimulate rapid IGF-1R signaling through ERα and IGF-1R may also activate ERα through ERK1/2 (52). These data support an amplification of IGF-1R and ERα signaling through their cross-activation of one another. Interestingly, IGF-1R expression has been shown to be increased in response to progesterone in normal endometrium complicating the understanding of each one’s role in the EMT of EC (48).

#### High glucose and diabetes

Impaired glucose metabolism and diabetes are risk factors for the development of endometrial cancer (17), and diabetes significantly increases the risk of death in patients with endometrial cancer (53). In addition to the role of these conditions in creating insulin resistance and increasing signaling through the insulin and IGF-1R pathways, elevated glucose levels themselves may contribute to the EMT of endometrial through other, often interrelated, mechanisms. Endometrial cancer cells exposed to high glucose conditions *in vitro* show increased proliferation and EC tissues from patients with diabetes exhibit mesenchymal biomarkers (decreased E-cadherin and increased N-cadherin) (54). One mechanism believed to cause these changes is activation of dynamin-related protein 1 (Drp1), the key protein in mitochondrial fission, in the setting of high glucose. Drp1 is increased in endometrial cancer patients with diabetes, and high glucose activates Drp1 in endometrial cancer to induce mitochondrial dysfunction, cellular progression through the cell cycle, increased migration and invasion, and expression of an EMT phenotype (decreased E-cadherin; increased N-cadherin, vimentin, and SNAIL) (54).

A unique form of post-translational protein modification, β-N-acetylglucosaminylation (O-GlcNAcylation), may also provide a link between high glucose states and EMT in endometrial cancer. O-GlcNAcylation is the process of adding N-acetylglucosamine, a glucose derivative and byproduct of the hexosamine biosynthesis pathway, to Serine and Threonine residues on proteins in a mechanism known to be responsive to nutrient availability. Altered levels of O-GlcNAcylation have been associated with several chronic conditions, including type II diabetes. Importantly, in type II diabetes, increased O-GlcNAcylation occurs in certain tissues, such as skeletal muscle and endothelial cells, and may contribute to insulin resistance (55, 56). Endometrial cancers have a high incidence of mutations in the genes encoding the O-GlcNAc cycling enzymes, O-GlcNAc transferase (OGT) and O-GlcNAcase (OGA). Moreover, O-GlcNAcylation is upregulated in some endometrial cancer cell lines and hyper-O-GlcNAcylation is associated with increased migration, a mesenchymal morphology and increased expression of the EMT marker N-cadherin. On the other hand, hypo-O-GlcNAcylation is associated with decreased proliferation and decreased expression of certain EMT-related genes, such as TGFB2. Complicating a clear understanding of the role of O-GlcNAcylation in the EMT of endometrial cancer, hyper-O-GlcNAcylation is associated with increased E-cadherin and decreased SNAIL expression, and O-GlcNAcylation status had no impact on the expression of the EMT markers Vimentin, SLUG, and ZEB1 (57). These findings highlight the need for further investigation into the role of the metabolically responsive O-GlcNAcylation process in the EMT of endometrial cancer.

A further mechanism by which high glucose states may induce EMT in endometrial cancer is upregulation of the insulin-controlled glucose transporter, GLUT4. Endometrial cancer tissues have increased expression of GLUT4 compared to the normal endometrium. Moreover, there is evidence that high glucose states increase expression of the estrogen receptors ERα and ERβ, which act on GLUT4 to stimulate VEGF/VEGFR mediated EMT by upregulating the EMT-associated genes TWIST, SNAIL, and CTNNB1 (58). This mechanism is particularly interesting as it shows the interplay between sex hormone signaling and impaired glucose metabolism in contributing to the EMT of endometrial cancer.

#### Metformin

Providing an additional level of support for the role of high glucose, insulin resistance, and diabetes in the role of EMT in endometrial cancer, the common diabetes treatment, metformin, has been shown to inhibit the EMT process. Tumor tissues from diabetic endometrial cancer patients on metformin show increased expression of E-cadherin. Additionally, *in vitro* studies reveal that metformin decreases endometrial cancer cell migration and invasion and influences epithelial marker expression with increased E-cadherin and decreased N-cadherin, vimentin, and fibronectin (59, 60). Furthermore, metformin downregulates the EMT transcription factors TWIST1, SNAIL, and ZEB1 in some endometrial cell lines (59). Some data suggests that metformin produces this effect by downregulating the PI3K/AKT/MDM2 pathway (60) and others have shown that metformin can inhibit 17β-estradiol stimulated EMT through ERK1/2 (24). Metformin may therefore provide yet another link between estrogen signaling and insulin resistance in the EMT of endometrial cancer.

## Discussion

While the associations of endocrine disorders including obesity, insulin resistance, and diabetes with endometrial cancer have long been recognized, the growing body of literature describing the role of endocrine disorders in the epithelial mesenchymal transition of endometrial cancer support the ongoing role of these conditions in the progression and metastasis of endometrial cancer even after they may have contributed to its carcinogenesis. Moreover, the interconnected actions of estrogen, insulin, IGF-1, and hyperglycemia on the EMT of endometrial cancer may explain the increased mortality rates seen in endometrial cancer patients with obesity and diabetes (53, 61), as advanced stage is associated with significant decreases in survival in endometrial cancer (1).

Though the above data provide compelling evidence for the role of multiple related endocrine pathways in driving the epithelial mesenchymal transition in endometrial cancer and many provide possible explanations for noted clinical trends, they are limited by the fact that the majority of studies have been conducted *in vitro* using endometrial cancer cell lines. *In vivo* studies using xenograft and transgenic models of endometrial cancer are needed to validate the findings observed in cell lines.

Limited clinical data from applications of these endocrine related EMT processes in endometrial cancer are mixed. For example, randomized control trial data show that treatment with metformin plus megestrol acetate for fertility sparing management of atypical endometrial hyperplasia and endometrial cancer may improve early complete response in hyperplasia patients but does not improve response in endometrial cancer patients (62). Conversely, diabetic endometrial cancer patients taking metformin experience improved survival (63). Data linking improved outcomes in endometrial cancer with metformin treatment would support the role of multiple endocrinological disturbances in the EMT of endometrial cancer, as metformin has been shown to counteract the effects of elevated estrogen levels, hyperglycemia and insulin resistance as detailed above. Tumor tissue analysis, or analysis of circulating tumor cells, with comparison of pre- and post-treatment clinical samples, is needed to determine whether endocrine targeted therapy can modulate or reverse the EMT phenotype. Future studies should be directed towards further *in vivo* confirmation of these findings and clinical applications that may alter the course of endocrine-mediated epithelial mesenchymal transition in endometrial cancer.

## Author contributions

JG: Investigation, Writing – original draft preparation, review & editing. GH: Supervision, Writing – review & editing. All authors contributed to the article and approved the submitted version.

## Conflict of interest

The authors declare that the research was conducted in the absence of any commercial or financial relationships that could be construed as a potential conflict of interest.

## Publisher’s note

All claims expressed in this article are solely those of the authors and do not necessarily represent those of their affiliated organizations, or those of the publisher, the editors and the reviewers. Any product that may be evaluated in this article, or claim that may be made by its manufacturer, is not guaranteed or endorsed by the publisher.
